# Influence of electron beam irradiation on water-saturated biodiesel

**DOI:** 10.1007/s10967-018-6153-1

**Published:** 2018-08-24

**Authors:** Paweł Grabowski, Przemysław Jarosiński, Piotr Szajerski, Hanna Gwardiak

**Affiliations:** 1 Institute of Chemistry, Faculty of Civil Engineering, Mechanics and Petrochemistry, Warsaw University of Technology, 17 Łukasiewicza Street, 09-400 Plock, Poland; 2 Department of Chemistry, Lodz University of Technology, 116 Zeromskiego Street, 90-924 Lodz, Poland; 3 Industrial Chemistry Research Institute, 8 Rydygiera Street, 01-793 Warsaw, Poland; 4 Present Address: Laboratory Masdiag, 27 Orzycka Street, Warsaw, Poland

**Keywords:** Biodiesel, E-beam irradiation, Biodiesel modification, Spectroscopy, Esters content

## Abstract

The objective was to study changes in water-saturated biodiesel irradiated by electron beam and to analyse them considering the influence of absorbed dose. Based on obtained results it can be concluded that irradiation did not affect ester groups in FAME molecules, but strongly influenced on double bonds. Total ester content decreased linearly with the increase in absorbed dose, causing FAME not to meet the requirement of PN-EN 14214 concerning the ester content (96.5 wt%). Therefore, the use of ionizing radiation to improve biodiesel properties is unlikely, but it is worth to consider electron beam sterilisation of this biofuel.

## Introduction

According to the recommendations of Directive 2009/28/EC of the European Parliament and of the Council, until 2020 European Union member countries are obliged to achieve 10% share of biofuels in general use of petrol and diesel fuel in transport [1]. EU requirements, combined with the awareness of finite natural resources and care for the environment, create need for research and development of new technologies for producing and improving biofuels.

Biofuels are gas or liquid products of biomass conversion, which is fundamental renewable energy source. Biomass is a general term for organic matter of living organisms. Substrates for obtaining biofuel are e.g.by-products generated by various kinds of plants, animals and in agriculture and forestry, industrial waste based on living organisms and crops cultivated to produce biofuel [2]. Generally, depending on raw material used, biofuels can be divided as following:

- first generation biofuels, obtained from edible crops,
- second generation biofuels, derived from uneatable resources,
- third generation biofuels, produced from algae [3].

Among biofuels that can be classified into each of above groups there is biodiesel. Biodiesel is the name for a variety of ester-based oxygenated fuels (and also their blends with diesel fuel) for compression-ignition engines. Chemically, biodiesel consists of monoalkyl esters of long-chain fatty acids derived from renewable biolipids. Biodiesel is obtained in transesterification, which is the reaction of triglycerides with a short-chain alcohol, usually methanol or ethanol. Due to exchange of alkoxy groups fatty acid alkyl esters are formed, as well as glycerol as a by-product [4].

Biodiesel, apart from the obvious advantages (e.g. high flash point [4], high biodegradability [5] and low PM, HC and CO emissions [6]), has some drawbacks. The most important are oxidation stability problems and resulting negative effect on plastics and other engine elements [7]. Such disadvantages make searching for biodiesel improvement necessary. One of possible techniques worth of consideration may be ionizing radiation.

Different energetic radiation types can induce chemical reactions or physical processes in materials. These types of radiation include photons (UV, X-rays) and particulate radiation (alpha, beta-electrons, e-beams and neutrons) [8]. In the literature a lot of information can be found, concerning overview on the principles of radiation sources, the physics of energy absorption processes and the resulting radiation effects in organic materials and polymers where the reactions are often referred to as ‘radiation chemistry’, i.e. the chemical changes and reactions induced by the deposition of radiation energy [8–16]. The most commonly available radiation types used in industrial processing are electron (e-beam) and gamma irradiation. E-beam generators have the additional advantage—they can be turned on and off. The ^60^Co gamma source may be available as a cheap radiation source but requires protection efforts.

^60^Co and e-beam sources induce similar changes in materials and can be used to irradiate different materials. As was described by Leathers et al. [8, 17] metals and inorganic substances simply absorb energy, undergo some fatigue processes or display defect formation, organic materials in comparison undergo specific radiation chemical reactions (i.e. the breaking and reforming of chemical bonds). The radiation chemistry involves bond cleavage and bond formation (cross-linking, leading to larger molecules) in organic materials. Radiation induced reactions in organic materials involve a broad spectrum of processes, to name a few: detrimental and long-term polymer degradation, controlled curing of reactive resins initiated by e-beam, polymer modifications via radiation induced grafting reactions, surface modifications of biomaterials, radiation induced depolymerization which is positive when degradation and low molecular weights are required (i.e. recycling) and negative when uncontrollable materials degradation occurs, sterilization of materials, oxidation and rapid breakdown of organic contaminants (i.e. water purification), organic waste processing (scission and breakdown of molecules in sludge and solution), and many others [8, 13, 15, 16].

The electron beam has a relatively large irradiation area compared to a gamma ray and can significantly reduce a processing time, and it can also vary the energy. In case of gamma rays, emission energy of usually used nuclide ^60^Co is 1.17–1.33 MeV (average 1.25 MeV), and emission energy of nuclide ^137^Cs is 0.667 MeV which is constant [10].

In the literature there is a lot of information concerning the preparation of microorganisms for synthesis of biofuels or sterilisation materials for fatty acids methyl esters (FAME) production, such as algae [18–22]. During this processes structure of fatty acid chain can be changed, which will cause change in biodiesel properties.

The aim of this work is to study changes in composition of water-saturated biodiesel irradiated by electron beam and to analyse them considering the influence of absorbed dose.

## Experimental

Considering the aspects of biodiesel storage, it can be noted that maintaining conditions completely deprived of water is very difficult. Due to the fact that water can significantly affect processes taking place in its presence as well as the biofuel itself, this work investigates the biodiesel-water system. Given the very limited miscibility of these substances, the samples were irradiated after being saturated with water. It is therefore expected that water disintegration products play an important role in the biodiesel radiolysis process.

The subject of this study was biodiesel derived from rapeseed (Rapeseed Methyl Esters, RME). After saturation RME with water, 10 samples were prepared, each containing 5 ml of biofuel. They were irradiated using linear electron beam accelerator Elektronika ELU-6e. The dosimetry was performed with the use of alanine dosimeters and Bruker EPR e-scan Alanine Dosimeter Reader. Absorbed doses of all samples are presented in Table 1.

**Table 1:** Doses of radiation absorbed by samples

Sample no	1	2	3	4	5	6	7	8	9	10
Absorbed dose (kGy)	1.07	2.87	5.22	10.37	20.79	40.61	60.31	80.91	100.14	245.90

After irradiation, ultraviolet–visible (UV–Vis) and infrared (IR) spectra of the samples were recorded. In the study spectrophotometers PerkinElmer LAMBDA™ 750 UV/Vis/NIR and Mattson Genesis II FT-IR were used. Composition of samples was examined with the use of gas chromatograph Agilent 7890A with selective mass detector 5975C, according to PN-EN 14103 [23].

## Results and discussion

### UV–Vis spectroscopy

**Fig. 1:**
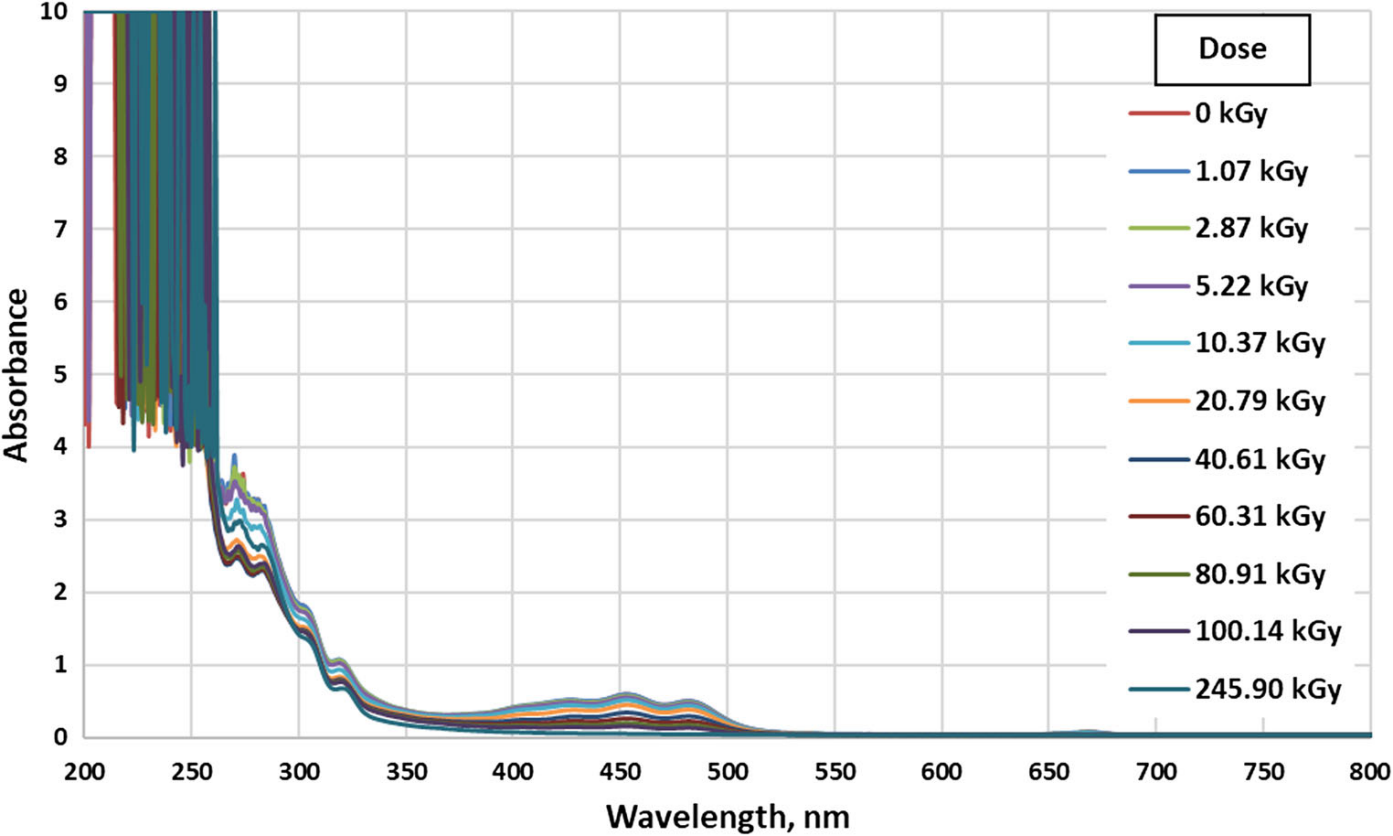
UV–Vis absorption of all tested samples

Figure 1 presents UV–Vis spectra of all samples. The ‘0 kGy’ label relates to nonirradiated reference sample.

**Fig. 2:**
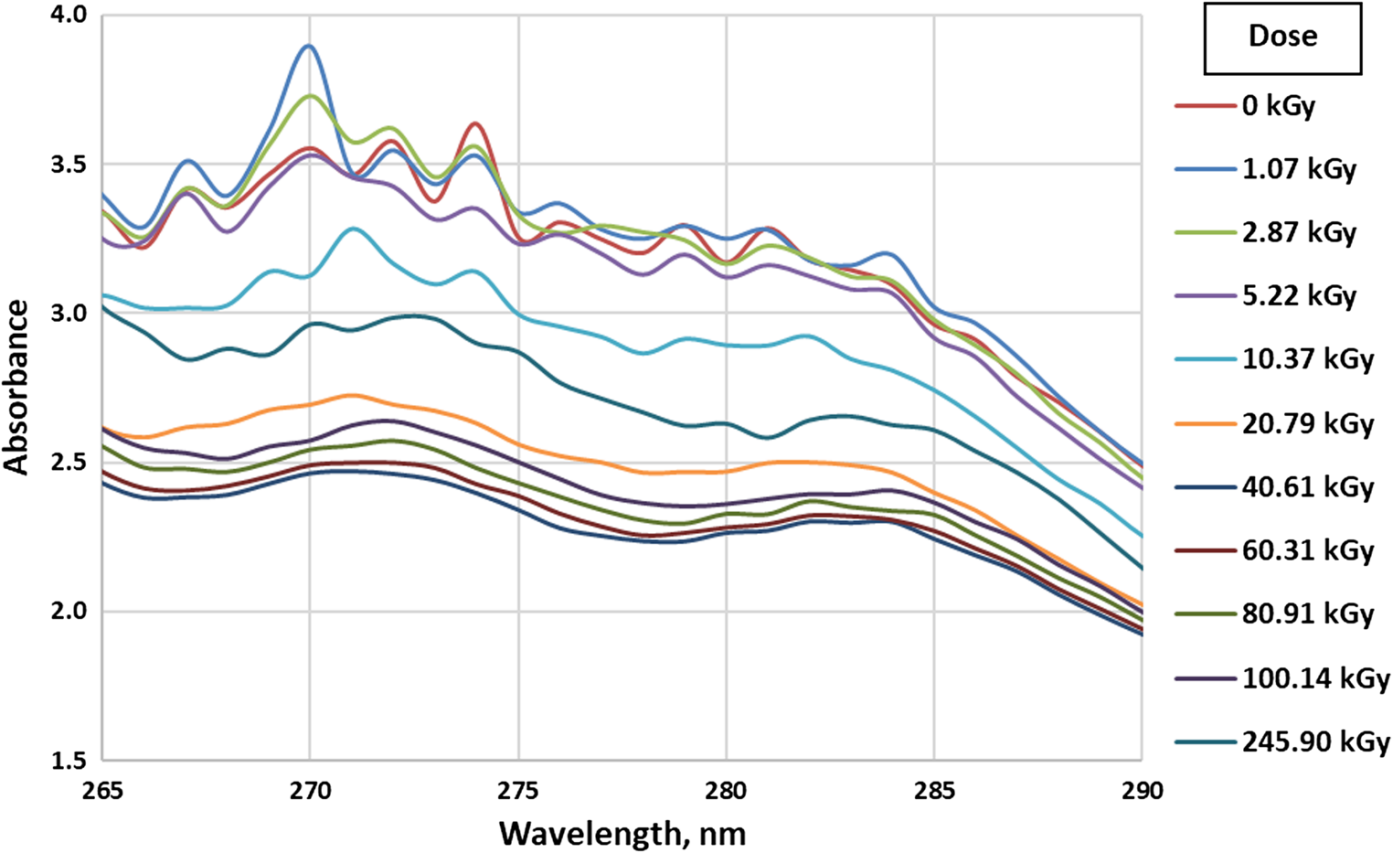
UV–Vis absorption of all tested samples in the range 265–290 nm

**Fig. 3:**
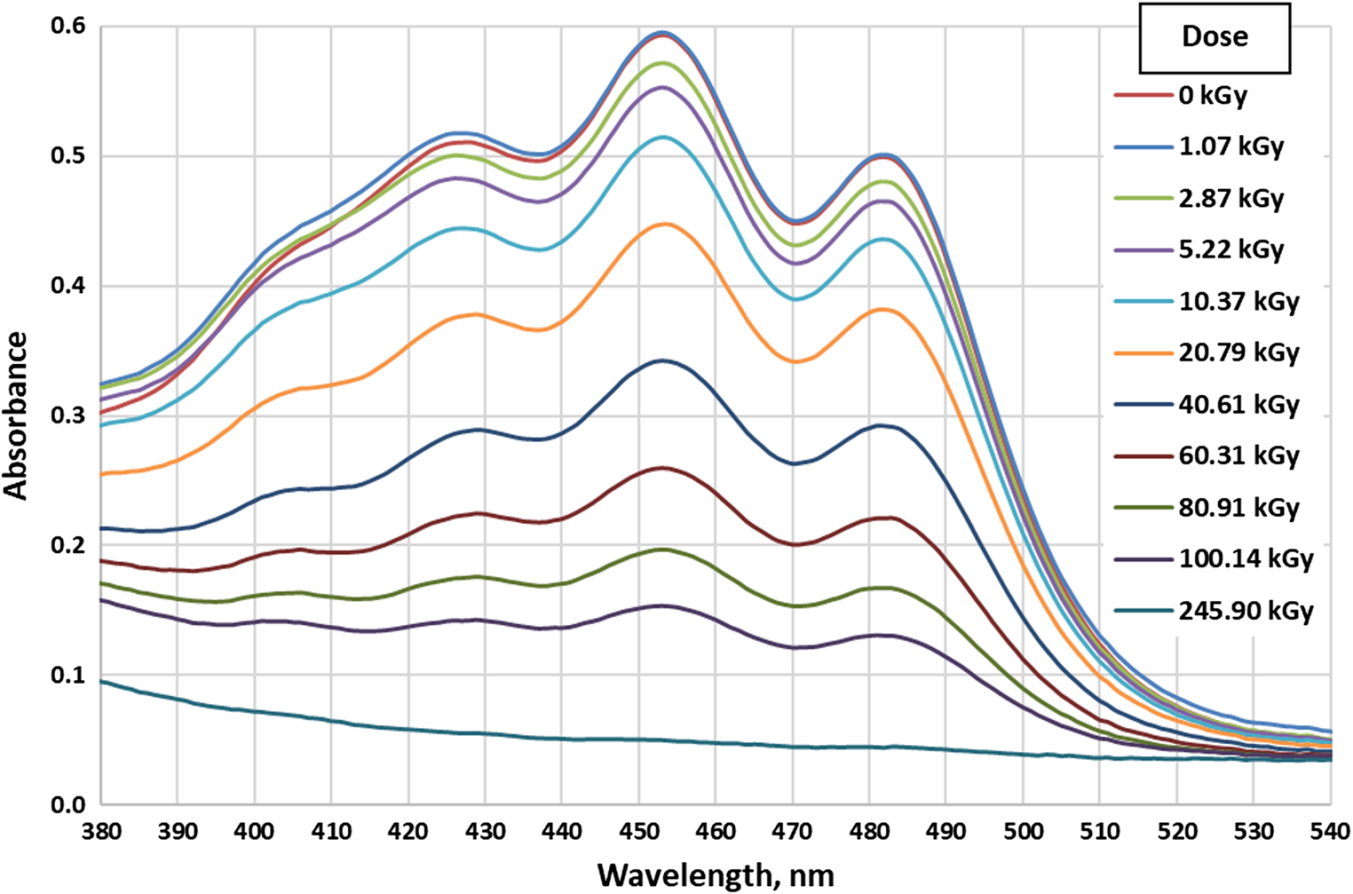
UV–Vis absorption of all tested samples in the range 380–540 nm

As can be seen in Fig. 1, changes that are possible to interpret occur in chromophores absorbing in the range 265–290 nm (Fig. 2) and 380–540 nm (Fig. 3). Other ranges can be neglected or there are no observable changes.

Radiation in the wavelength range from 200 to 800 nm is absorbed mainly by double bond systems. They can include from several to over a dozen bonds, depending on the compound [24].

As shown in Fig. 2, for the samples irradiated by the smallest doses (from 0 to 5.22 kGy) there is no significant change in absorption curves. At higher doses, as the dose increases to 40.61 kGy, the absorption decreases and then remains at approximately the same level. There can be two maxima distinguished—the first at about 270–272 nm and the second at about 282–284 nm. Absorption in this range occurs due to the transfer of electrons from bonding π to antibonding π* orbitals in conjugated systems of several double bonds [24]. Unsaturated esters are probably responsible for the absorption, but it is also possible that pigments present in biodiesel are involved. Regardless of the actual explanation, it can be concluded that due to ionizing radiation a reduction in the number of double bond systems occurs.

In the range 380–540 nm presented on Fig. 3 there are two very clear absorption maxima at the wavelengths of 453 and 482 nm. Conjugated unsaturated bond systems exhibit absorption in visible light, e.g. β-carotene containing 11 double bonds has the absorption maximum at 445 nm [24].

In the presented visible range, there is clear correlation between the absorption intensity and the dose absorbed by samples. With the increase in dose, the absorbance decreases, i.e. the amount of carotenoids present in biodiesel is reduced due to the destruction or transformation of unsaturated bond systems. Similar phenomena may occur in biodiesel itself.

### IR spectroscopy

**Fig. 4:**
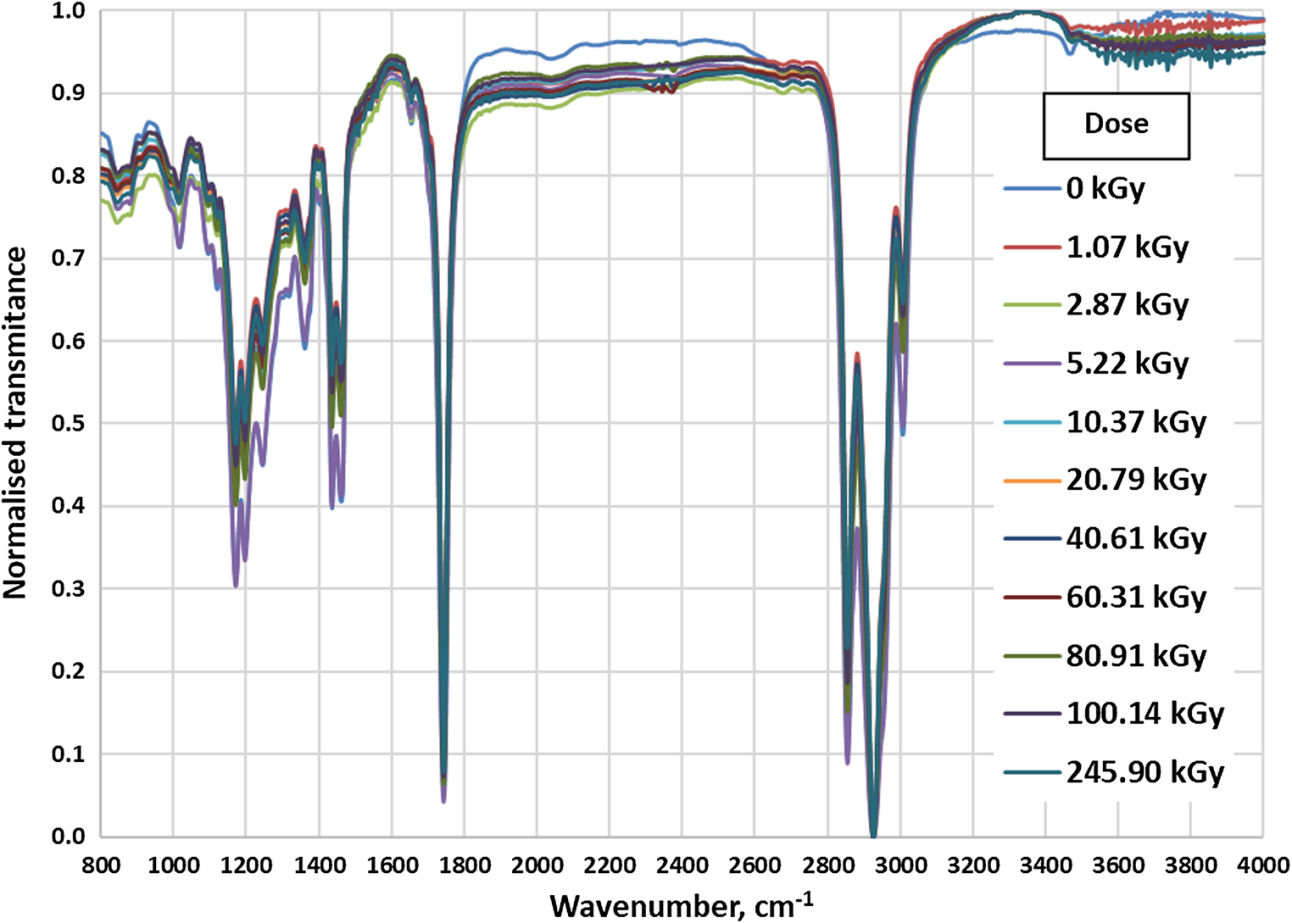
IR absorption of all tested samples in the range 800–4000 cm^−1^

For a more comprehensive study, infrared spectroscopy was also used. IR spectra were normalised and presented in Fig. 4.

Tested RME samples consist of many esters of different fatty acids and methanol. Esters have long hydrocarbon chains (often containing unsaturated bonds) and functional ester groups –COO–, which causes overlapping of many bands. Therefore, the interpretation of the results is difficult.

In the range 1000–1600 cm^−1^ there are many bands corresponding to vibrations of C–H and C–O bonds, but they are hard to distinguish. Similar situation occurs in the range 2800–3200 cm^−1^, where a few peaks of C–H vibrations can be observed [24].

**Fig. 5:**
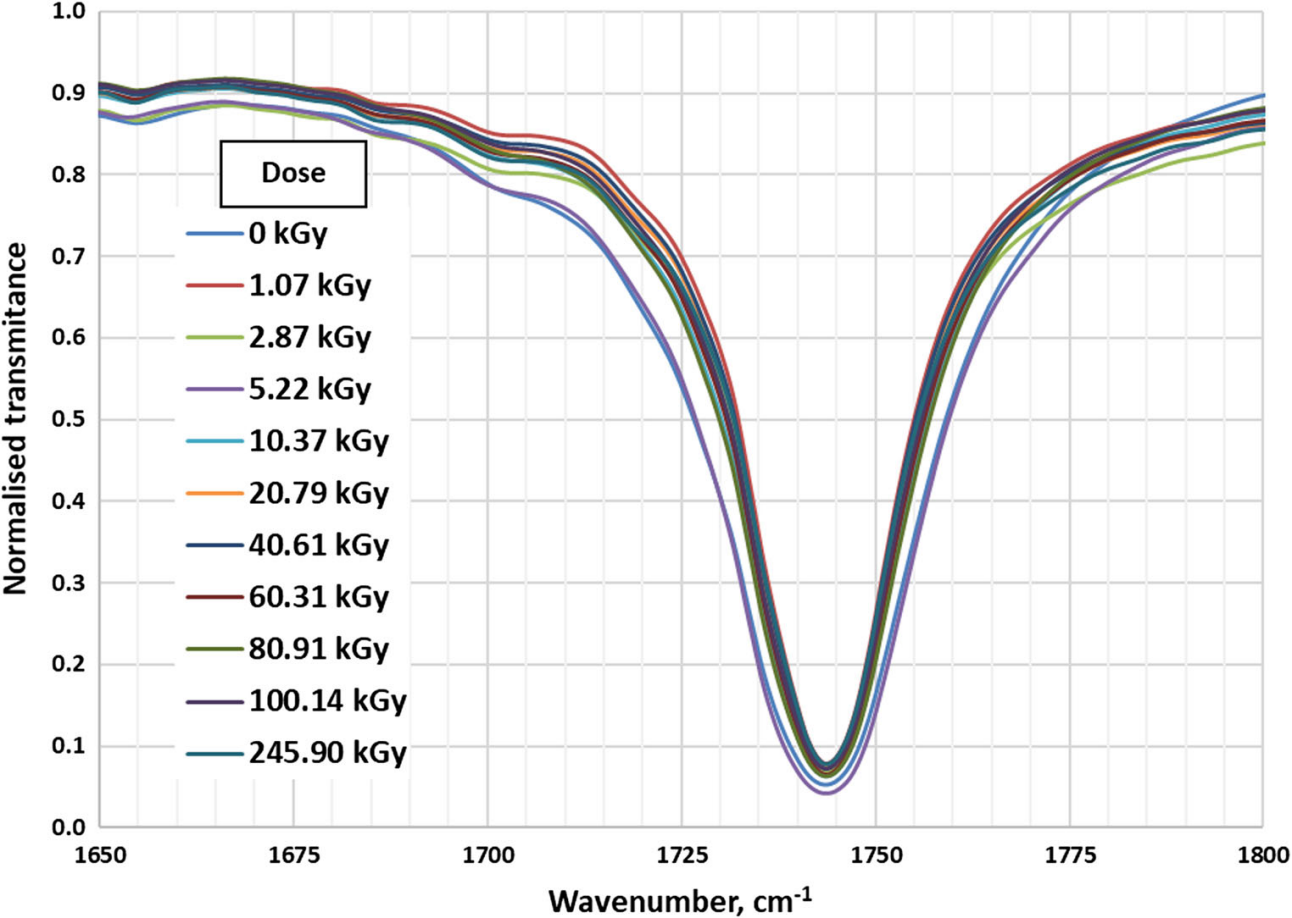
IR absorption of all tested samples in the range 1600–1800 cm^−1^

The clearest band present in discussed IR spectra is the peak at 1745 cm^−1^(Fig. 5). It corresponds to stretching vibrations of carbonyl group (C=O) in esters [24]. Intensity and location of the band do not depend on the absorbed dose of radiation. This means that ionizing radiation does not affect the carbonyl group in the esters in tested biodiesel samples.

### Composition of samples (ester content)

In order to accurately investigate changes in the composition of irradiated RME samples, gas chromatography was used. Measurements were made in accordance with the requirements of PN-EN 14103 [23]. Determined content of all esters present in biodiesel samples is shown in Table 2. The number accompanying ‘C’ letter represents the total quantity of carbon atoms in the ester chain, an additional number indicates the amount of unsaturated bonds.

**Table 2:** Ester content in all tested RME samples

	Dose of radiation (kGy)										
	0.00	1.07	2.87	5.22	10.37	20.79	40.61	60.31	80.91	100.14	245.90
Content of particular methyl esters (wt%)											
C14:0^a^	0.13	0.13	0.13	0.13	0.13	0.13	0.13	0.13	0.13	0.13	0.13
C16:0	4.84	4.78	4.79	4.79	4.78	4.77	4.76	4.74	4.75	4.73	4.67
C16:1	0.65	0.64	0.64	0.64	0.64	0.64	0.64	0.63	0.63	0.63	0.62
C18:0	1.77	1.75	1.76	1.76	1.76	1.76	1.76	1.76	1.76	1.76	1.77
C18:1	59.55	59.14	59.17	59.16	59.08	58.97	58.71	58.45	58.45	58.24	57.03
C18:2	18.70	18.00	18.07	18.06	18.03	17.94	17.78	17.68	17.62	17.52	16.83
C18:3	8.22	7.64	7.68	7.67	7.69	7.64	7.54	7.47	7.38	7.35	6.99
C20:0	0.54	0.54	0.54	0.54	0.54	0.54	0.54	0.53	0.53	0.53	0.53
C20:1	1.27	1.27	1.27	1.28	1.28	1.27	1.26	1.26	1.26	1.26	1.23
C22:0	0.28	0.29	0.30	0.30	0.29	0.30	0.29	0.29	0.29	0.29	0.30
C22:1	0.44	0.44	0.44	0.44	0.44	0.44	0.44	0.44	0.43	0.43	0.43
C24:0	0.12	0.12	0.12	0.12	0.12	0.12	0.12	0.12	0.13	0.13	0.13
C24:1	0.12	0.12	0.12	0.12	0.12	0.12	0.12	0.11	0.12	0.12	0.12
Total ester content (wt%)	96.89	95.14	95.29	95.25	95.15	94.89	94.33	93.74	93.73	93.37	91.01

^a^C14:0—methyl mirystate, C16:0—methyl palmitate, C16:1—methyl palmitoleate, C18:0—methyl stearate, C18:1—methyl oleate, C18:2—methyl linoleate, C18:3—methyl linolenate, C20:0—methyl arachidate, C20:1—methyl eicosanoate, C22:0—methyl behenate, C22:1—methyl erucate, C24:0– methyl lignocerate, C24:1—methyl nervonate

As can be read from Table 2, esters which concentrations decreases most are those which content in RME is the highest. The radiation reacts with the main constituents of biodiesel (the so-called matrix of the sample), that is why esters at low concentrations are not affected.

**Fig. 6:**
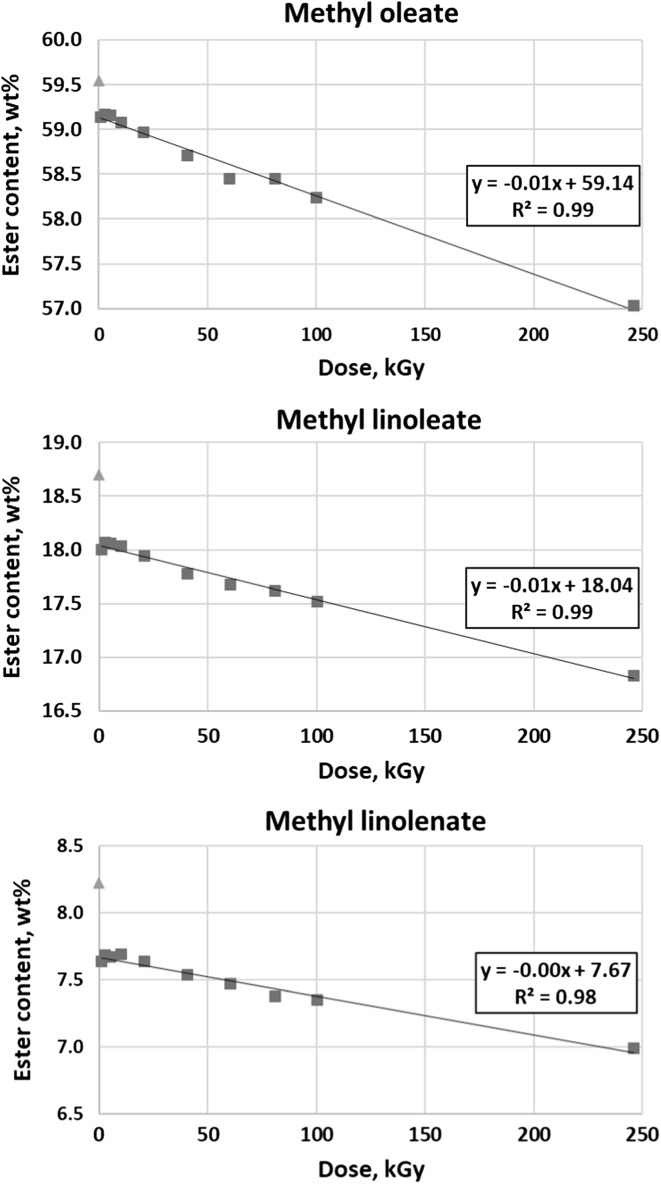
Content of unsaturated C18 esters versus dose of radiation

Electron radiation mostly influences on methyl oleate, methyl linoleate and methyl linolenate. Changes in concentration of these esters were presented in Fig. 6.

The most distinct difference in content of methyl oleate, as well as methyl linoleate and methyl linolenate is observed between the nonirradiated and 1.07 kGy sample. Further changes are not so significant considering the rate of increase in dose. Ester content and radiation dose are very well linearly correlated if the first measurement (nonirradiated sample) is excluded. The coefficient of determination (*R*^2^) values are 0.99 for methyl oleate and methyl linoleate and 0.98 for methyl linolenate.

**Fig. 7:**
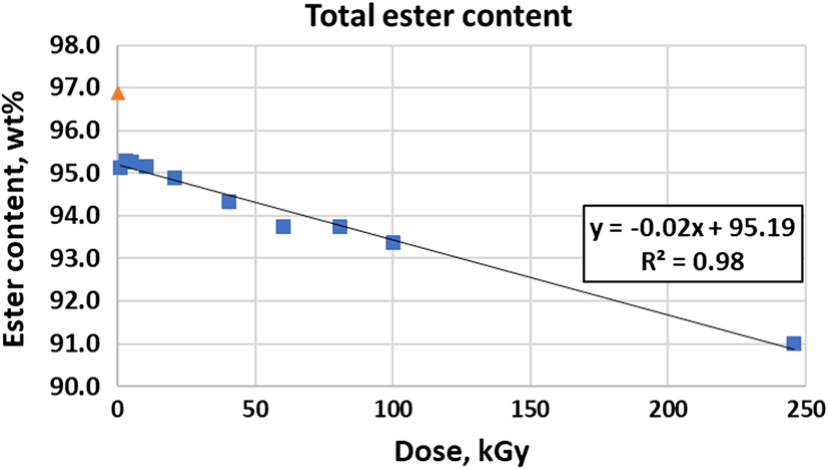
Total ester content versus dose of radiation

Changes in total ester content were presented in Fig. 7. The concentration decreases from 96.89 wt% in nonirradiated sample to 91.01 wt% in a 245.90 kGy sample. As in the case of C18 esters described above, the most significant decrease occurs from nonirradiated sample to 1.07 kGy sample. Also, the dependence on dose is clearly linear (*R*^2^ value is 0.98) if the nonirradiated sample is excluded.

Possible explanation of relatively small decrease in ester content may be that water present in samples absorbs most of the radiation and its radiolysis occurs. However, even the smallest dose causes biodiesel not to meet requirement of PN-EN 14214 which is 96.5 wt% [25].

### Dendrogram analysis

**Fig. 8:**
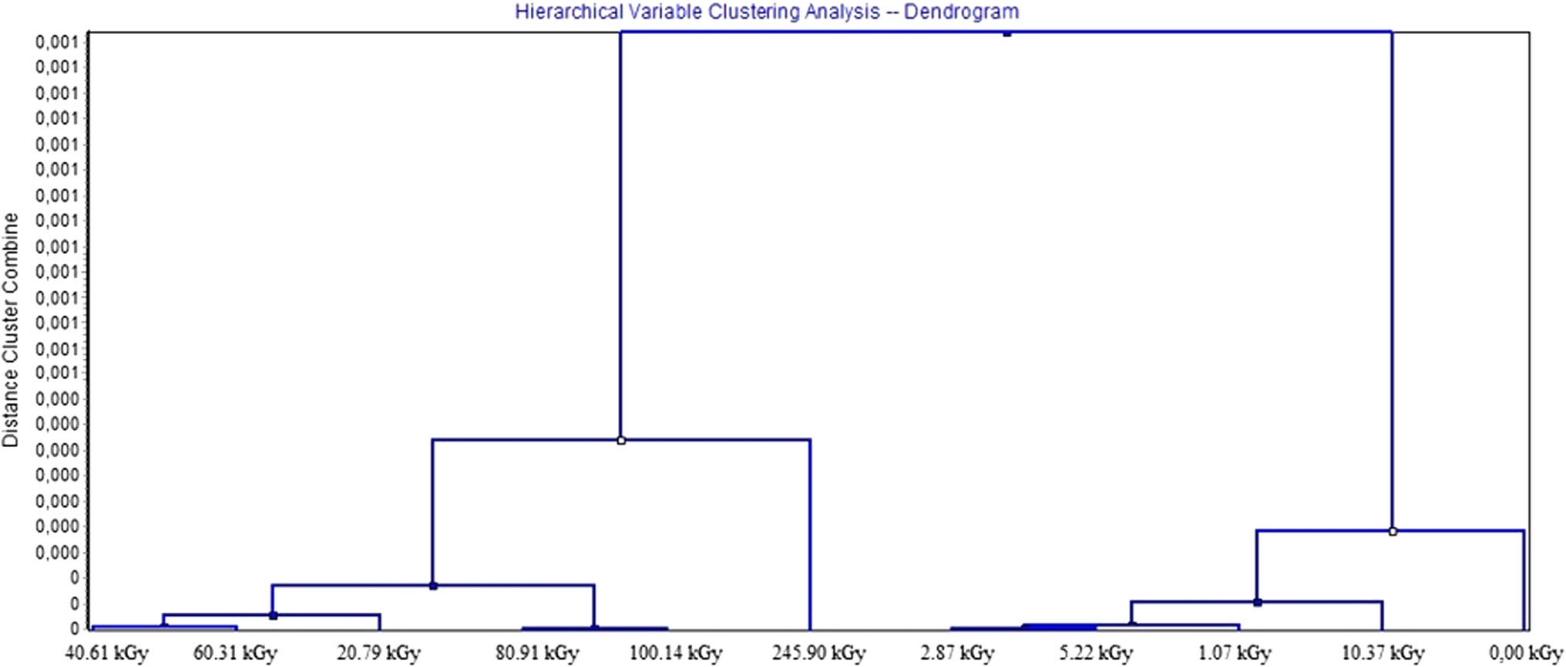
Cluster analysis for UV–Vis and GC result

Banding patterns generated for the data from analysis described in this article were used for cluster analysis and the creation of a dendrogram (Fig. 8). The dendrogram was performed using the TANAGRA software package [26]. The dendrogram was created using unweighted pair groupings of a similarity coefficient (*S*_AB_) matrix, and consistently resulted in the groupings shown in Fig. 8. On the basis of this analysis the samples were divided into two clusters. Each group corresponds to different changes in sample composition. The first group is associated with low radiation doses that directly connect to the nonirradiated sample. The second group consists of samples that have absorbed high doses of radiation. A sample exposed to the highest radiation dose may be considered as a separate group, but due to analogous variations such as 20–100 kGy dose samples, it was classified in the same group. Cluster analysis confirms observations resulting from direct analysis of data obtained from UV–Vis and GC studies. For low doses (up to about 10 kGy), statistical effects of radiation from RME are observed, while for high doses (from about 10 kGy) the results indicate that the polymerisation and/or degradation of methyl esters of higher fatty acids is present.

### Principal components analysis

PCA analysis was performed based on all measured concentrations of fatty acids methyl esters and the absorption maxima in the range UV–Vis in FAME samples treated with ionizing radiation. According to the PCA analysis, the three main components contain 88.12% of the information. PCA1 (69.84%) and PCA2 (10.47%) as shown in the figure cover as much as 80.31% of the information contained in the measurement data. Based on this information, a PCA analysis can be applied to the description of the examined dataset.

**Fig. 9:**
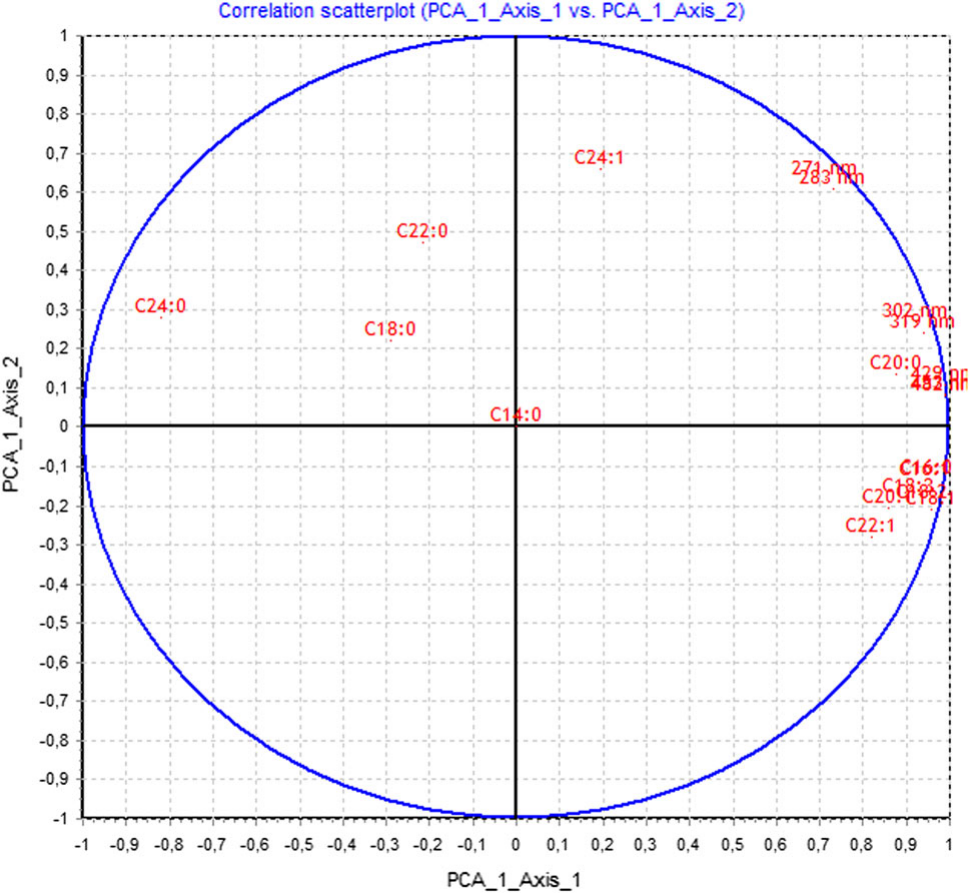
PCA analysis plot

The purpose of the analysis was to identify the relationship between the change in absorbance and the concentration of methyl esters. As presented in the graph shown in Fig. 9, changes in absorbance in the UV–Vis range are not related to changes in saturated ester concentrations such as C14:0, C18:0, C22:0 and C24:0. Changes in absorbance correlate with changes in esters containing unsaturated bonds (double and triple), i.e. they confirm the effects of ionizing radiation with polyunsaturated fatty acids (PUFAs). These correlations confirm the presence of all wavelengths and ester concentrations on the right side of the graph in Fig. 9.

## Conclusions

On the basis of conducted research, it can be concluded that ionizing radiation causes changes in composition of rapeseed methyl esters. Spectroscopic methods can be used to preliminary study these changes. Infrared spectroscopy results suggest that carbonyl groups in esters are not affected by radiation. Analysis in ultraviolet–visible range indicates that electron beam mainly influences on unsaturated bonds in ester chains. However, the UV–Vis spectroscopy results may be affected by the presence of carotenoids in biodiesel. Carotenoids, containing conjugated double bond systems, are very sensitive to ionizing radiation. The bonds can be destroyed or transformed due to reactions like saturation or polymerisation. Similar processes probably occur in unsaturated esters molecules. The reduction of double bonds can cause changes in macroscopic parameters of biodiesel, like low-temperature properties. Larger volumes of samples should be irradiated to examine the possible changes.

Determination of ester content with the use of gas chromatography seem to correspond with spectroscopic results. The observed decrease in concentration of unsaturated esters, combined with IR conclusion concerning no changes in carbonyl group absorption, suggests that double bonds may undergo fragmentation, polymerisation or saturation while the carbonyl group in esters remains intact.

Esters most affected by electron radiation are methyl oleate, methyl linoleate and methyl linolenate. These are compounds which concentration in tested biodiesel is the highest. The most distinct change in content is in each case observed between the nonirradiated sample and the sample irradiated with 1.07 kGy. Further decrease is always linear with very good coefficient of determination (*R*^2^), which is 0.98 or 0.99. The results of total ester content are similar, with analogous *R*^2^ (0.98). Total ester content decreases from 96.89 wt% in nonirradiated sample to 91.01 wt% in a 245.90 kGy sample. This relatively small (considering very high doses used) change can be explained by probable absorption of radiation by water present in samples. Unfortunately, even the least irradiated sample does not meet the requirement of PN-EN 14214 concerning the ester content (96.5 wt%). Therefore, the use of ionizing radiation to improve RME properties is questionable. However, instead of modifying biodiesel, sterilising it with the use of electron beam is worth considering because the sterilisation doses are much smaller [27] and the problem of microbial growth in fuel is still very important issue.
